# Effects of ondansetron treatment on outcomes of critically ill patients with myocardial infarction partly through its anti-inflammatory activity

**DOI:** 10.7150/ijms.81797

**Published:** 2023-04-17

**Authors:** Yang Boshen, Zhu Yuankang, Li Taixi, Niu kaifan, Wang Zhixiang, Liu Liang, Shen Chengxing, Lu Xia, Jin Xian

**Affiliations:** 1 Department of Cardiology, Shanghai Sixth People's Hospital Affiliated to Shanghai Jiao Tong University School of Medicine, Shanghai, China; 2 Institute for Developmental and Regenerative Cardiovascular Medicine, Xinhua Hospital, 12 School of Medicine, Shanghai Jiao Tong University, Shanghai, China

**Keywords:** Hospital mortality, inflammation, myocardial infarction, ondansetron, treatment

## Abstract

**Background:** Patients with myocardial infarction (MI) in intensive care units (ICU) are at high risk of death. Whether treatment with ondansetron (OND) at an early stage plays a protective role in critically ill patients with MI and its underlying mechanism remains unclear.

**Methods:** A total of 4486 patients with MI were enrolled in the study cohort from the Medical Information Mart for Intensive Care IV (MIMIC-IV) database and divided into OND-medication groups or not. Propensity score matching (PSM) and regression analysis were performed to investigate the effect of OND on patients, accompanied by sensitivity analysis to evaluate the robustness of the results. Integrated with causal mediation analysis (CMA), we investigated the potential causal pathway mediated by the palate-to-lymphocyte ratio (PLR) between early OND treatment and clinical outcomes.

**Results:** Among patients with MI, 976 of them were treated with OND at the early stage while 3510 patients were not. The all-cause in-hospital mortality rate was significantly lower in the OND-medication group (5.6% vs 7.7%), accompanied by lower 28-day mortality (7.8% vs 11.3%) and 90-day mortality (9.2% vs 13.1%) rates. PSM analysis further confirmed the results for in-hospital mortality (5.7% vs 8.0%), 28-day mortality (7.8% vs 10.8%), and 90-day mortality (9.2% vs 12.5%). After adjusting for confounders, multivariate logistic regression analysis revealed that OND was associated with decreased in-hospital mortality (OR = 0.67, 95% CI: 0.49-0.91), and Cox regression confirmed the results for 28-day mortality and 90-day mortality with HR = 0.71 and 0.73, respectively. Most importantly, CMA demonstrated that the protective effect of OND on patients with MI was mediated by its anti-inflammatory effect through the regulation of PLR.

**Conclusion:** Early use of OND in critically ill patients with MI may exert protective effects by reducing in-hospital mortality and 28- and 90-day mortality. The beneficial effects of OND on these patients were exerted through anti-inflammatory effects, at least in part.

## Introduction

Myocardial infarction (MI) is a prevalent health problem with high morbidity and mortality, usually caused by prolonged ischemia and hypoxia of the heart^1^. The adverse clinical outcomes of patients with MI have been improved owing to the rapid development of percutaneous coronary intervention^2^. However, in intensive care units (ICU), patients frequently deal with significant complications, accompanied by several acute or chronic cardiac and non-cardiac conditions^3^,^4^. Therefore, it is urgent to identify feasible measures to reduce the mortality of critically ill patients with MI.

Inflammation plays an indispensable role in cardiovascular diseases (CVDs). The involvement of the immune system has been well recognized as a major component of atherosclerosis, a chronic inflammatory disease, and MI^5^. Several inflammatory chemokines and cytokines, such as interleukin-1 (IL-1) and tumor necrosis factor-α (TNF-α), promote immune cell infiltration^6^-^9^. Notably, suppressing myocardial inflammation post-MI could augment cardiomyocyte apoptosis, increase infarct size, and deteriorate cardiac function^10^.

Ondansetron (OND), a type of serotonin (5-HT_3_) receptor antagonist, has long been used to control nausea and vomiting caused by chemotherapy. Early use of OND may reduce the occurrence of complications and mortality rate by acting as an antiemetic drug^11^-^13^. Interestingly, accumulated evidence has suggested that OND may have anti-inflammatory effects. For instance, OND inhibited the expression of pro-inflammatory cytokines such as IL-1β, IL-6, TNF-α, and inducible nitric oxide synthase by targeting peritoneal macrophages in an intestinal manipulation animal model^14^ and attenuated pancreatic injury through its anti-inflammatory action in a cerulein-induced acute pancreatitis model^15^. In addition, it has recently been reported that platelet-derived 5-HT contributes to increased inflammation in the infarct area and reduced myocardial salvage, indicating that targeting the 5-HT receptor may be a potential treatment for ischemic heart disease^16^. Therefore, we hypothesized that OND exposure may provide protective effects for critically ill patients with MI through its anti-inflammatory activity.

However, despite the high drug tolerability, the cardiac side effect of OND for QTc prolongation has been observed in some high-risk patients^17^. Therefore, further consideration should be given to the possibility that OND may play a protective role in patients with CVD, especially those in the ICU.

In this study, we aim to investigate whether critically ill patients with MI can benefit from the use of OND during their ICU stay. Using causal mediation analysis (CMA), we further investigated whether the cardioprotective effect of OND was mediated by an anti-inflammatory effect.

## Materials and methods

### Data source and study design

The study participants and clinical indices were extracted from the Medical Information Mart for Intensive Care IV (MIMIC-IV) database^18^. The MIMIC-IV database is a large and high-quality public database consisting of electronic health records of patients admitted to the ICU or emergency department of the Beth Israel Deaconess Medical Center from 2008 to 2019, which has more than 40000 patients. After passing the qualification tests and obtaining the database access, SQL language and ICD-9 code were used to extract the required patients and relevant indices.

This is a large retrospective cohort study. All patients diagnosed with MI on hospital admission were included in the analysis based on the ICD-9 code. Patients with OND medication records within 24 hours before entering the ICU and within 72 hours after entering ICU were included in the OND-medication group, and the rest were defined as non-OND medication group. The exclusion criteria were as follows: 1 age < 18 years old and 2 have no ICU stay records. Multiple strategies were used to evaluate the protective effect of OND on patients with MI and potential confounders were considered in the analysis to avoid bias.

### Clinical variables

Vital signs, clinical indices, and complications of study participants were included. For vital signs, we extracted the first measured data within 24 hours after admission to the ICU. Similarly, for clinical indices, the initial measurement obtained on the first day after ICU admission was included. Additionally, the use of diuretics and vasopressin and some disease scores such as Sequential Organ Failure Assessment (SOFA) and Simplified Acute Physiology Score II (SAPS II), were also included in the analysis. Indices with missing values of more than 30% were deleted, and the rest were filled with multiple imputations.

### Primary and secondary outcomes

The primary outcome was defined as all-cause in-hospital mortality of patients with MI in the ICU. Furthermore, 28- and 90-day mortality were considered secondary outcomes. SOFA and SAPS II were also included as clinical outcomes. All study participants were followed up until death or 28 or 90 days following hospital discharge.

### CMA and sensitivity analysis

Palate-to-lymphocyte ratio (PLR) was used as a mediator for CMA to analyze the causal relationship between early OND medication and clinical outcomes. In CMA, the total effect of OND medication on in-hospital mortality was divided into direct and indirect effects, which were represented by the average direct effect (ADE) and average causal mediation effect (ACME), respectively. The ADE refers to the direct relationship between OND use and the primary outcome, not mediated by the mediator, keeping the mediated pathway fixed. ACME represents the indirect relationship between OND use and the outcome via the mediator. Compared with traditional correlation analysis, more in-depth conclusions were drawn. We assumed an unmeasured confounder to evaluate the robustness of CMA and performed a sensitivity analysis to examine the influence of the unmeasured confounder on the results. The unmeasured confounder was represented as ρ.

### Statistical analysis

Data were presented in the tables according to different distributions and types of variables. Categorical variables, such as sex and comorbidities, were presented as numbers (percentages) and tested by chi-square tests (or Fisher's exact). Continuous variables, such as blood pressure and platelet, were presented as mean ± standard deviation or median (25-75 percentiles) and were tested by student's t-test or Wilcoxon rank-sum tests.

Multivariate modeling of the relationship between OND medication and in-hospital mortality was investigated using a logistic regression model, and a Cox regression model was used to reveal the relationship between OND medication and 28-day or 90-day mortality. All potential confounders were considered in the multivariate regression models. The effect of OND medication was represented by odds ratio (OR) with a 95% confidence interval (CI) for in-hospital mortality while hazard ratio (HR) with a 95% CI for 28- and 90-day mortality.

Propensity score matching (PSM) analysis was used to balance potential confounders between the two groups. PSM method was performed without replacement based on a 1:1 matching ratio via the nearest neighbor, and a caliper width of 0.02 was applied in this study. All potential confounders were taken into consideration in the PSM method. Outcomes were regenerated following PSM, constructed with 974 patients in both groups.

All statistical analyses in this study were performed using SPSS (version 23.0) or Stata (version 14.0). A mediation package in State was adopted for CMA. A *P*-value <0.05 was set for statistical significance in this study.

## Results

### Baseline characteristics and clinical outcomes of two groups

**Figure 1:**
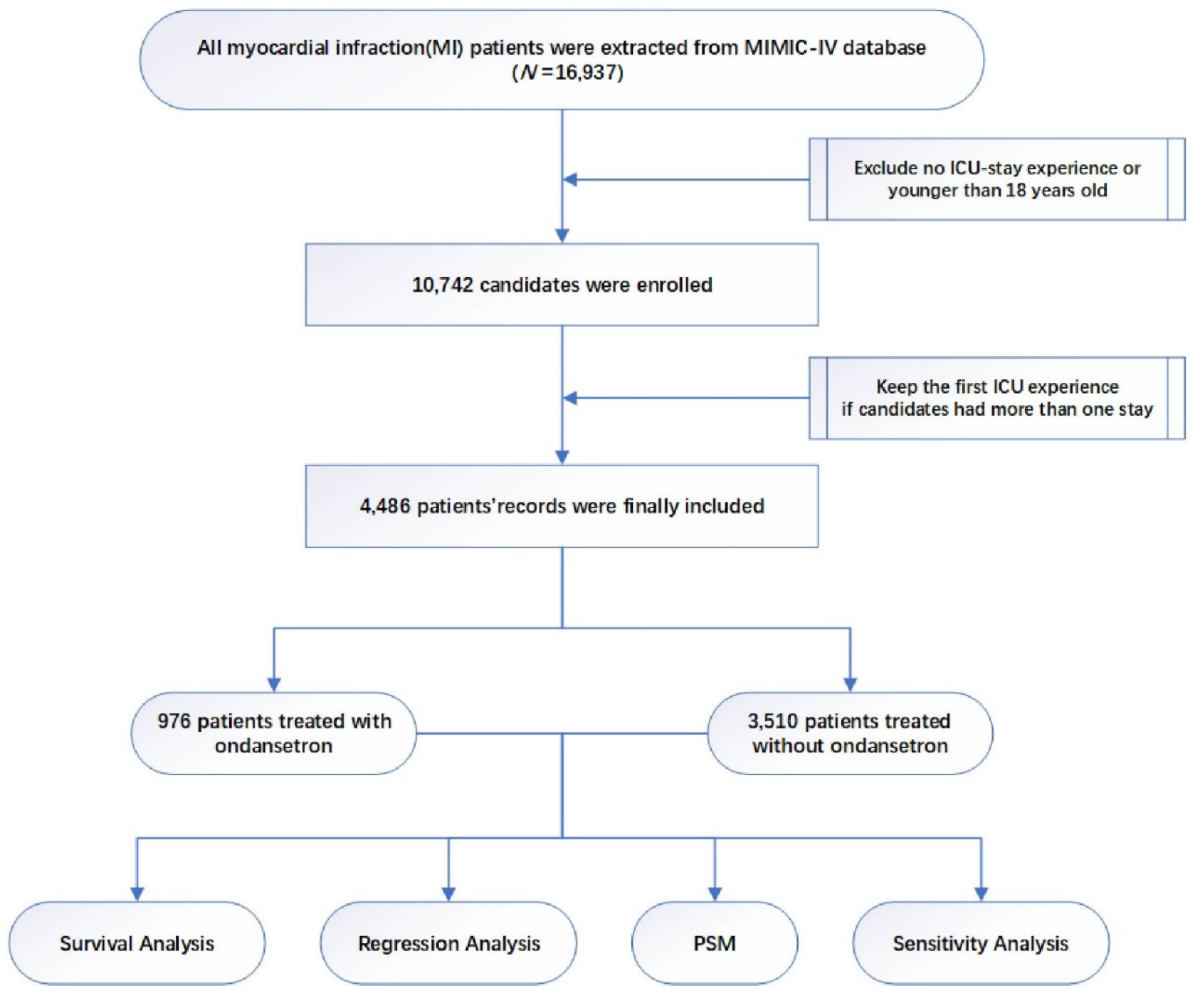
Study flowchart.

The study design is displayed as a flowchart in** Figure 1**. After the selection of patients, a total of 4486 critically ill patients with MI were enrolled in the final cohort. Among them, 976 patients were treated with OND at an early stage while 3510 patients were not. Baseline characteristics and clinical outcomes among patients using OND or not are presented in **Table 1**. Although a lower proportion of males were treated with OND in an early stage, no significant differences in age and weight were observed. There were no significant differences in systolic blood pressure, diastolic blood pressure, temperature, and HR; however, the OND-medication group had lower respiratory rate (RR). Higher incidences of chronic obstructive pulmonary disease (COPD), chronic kidney disease (CKD), acute kidney injury, and congestive heart failure (CHF) were observed in the non-OND medication group while the two groups had similar incidences of atrial fibrillation (AF) and cerebral hemorrhage. In terms of clinical data, patients treated with OND had higher PLR while there were no significant differences in lymphocyte, platelet, red blood cell (RBC), glucose, and white blood cell (WBC). A higher frequency of vasopressin use was observed in the non-OND medication group.

The primary outcome, in-hospital mortality, revealed that patients treated with OND had a lower risk of death compared with those not (5.6% vs 7.7%). Similarly, the OND-medication group had lower 28-day mortality (7.8% vs 11.3%) and 90-day mortality (9.2% vs 13.1%) rates.

### Association between early OND treatment and clinical outcomes in patients with MI

As presented in **Table 2**, in unadjusted model 1, the OR between OND and in-hospital mortality was 0.72 (95% CI: 0.53-0.97). After adjusting for sex and age, the association remained with an OR of 0.72 (95% CI: 0.53-0.97). To further avoid biases caused by potential confounders, model 3 was adjusted for age, sex, CHF, AF, COPD, CKD, cerebral hemorrhage, WBC, RBC, glucose, platelet and creatinine, and the protective effect of OND on patients with MI was still consistent with OR = 0.67 (95% CI: 0.49-0.91).

**Figure 2:**
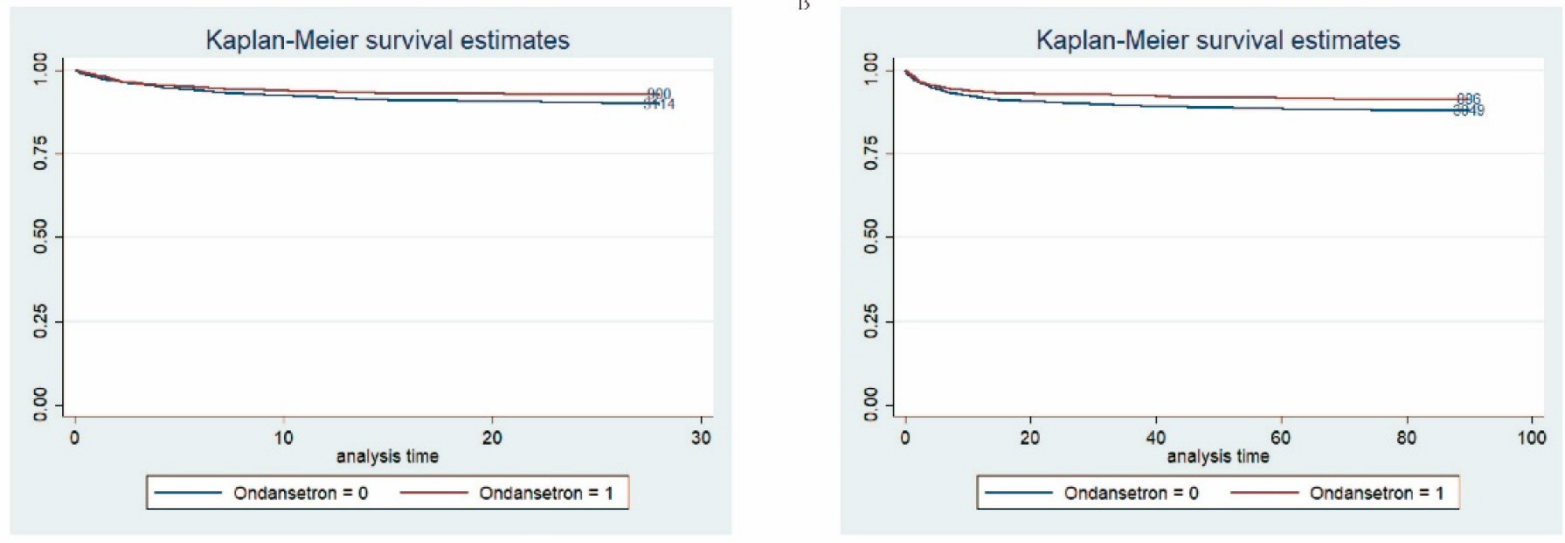
** Survival analysis.** (A) Kaplan-Meier curve between patients treated with OND at an early stage and those were not within 90 days. 1 represents patients treated with OND. (B) Kaplan-Meier curve between patients treated with OND at an early stage and those were not within 90 days. 1 represents patients treated with OND.

Cox regression analysis was applied to further investigate the impact of time on 28-day and 90-day mortality. As presented in **Table 2,** the HRs of OND medication for 28-day mortality and 90-day mortality were 0.71 (95% CI: 0.55-0.92) and 0.73 (95% CI: 0.58-0.92), respectively. As for survival analysis, Kaplan-Meier curve revealed that patients treated with OND had a lower risk of death and greater survival rates within 28 and 90 days (**Figure 2**). *P*-values for log-rank tests were lower than 0.05.

Additionally, patients with MI treated with OND at the early stage had lower SOFA [4(2-6) vs. 4(2-7)] and SAPS II [35(28-43) vs. 36(29-45)] during ICU stay compared with those not.

### Clinical outcomes after PSM

As presented in **Table 3**, 1948 patients, including 974 OND users, were matched by a 1:1 matching ratio after PSM. No significant difference was observed in all matched factors (*P* > 0.05). In the matched cohort, in-hospital mortality (5.7% vs 8.0%), 28-day mortality (7.8% vs 10.8%), and 90-day mortality (9.2% vs 12.5%) rates were significantly lower in patients treated with OND. In terms of disease severity, there were lower SOFA (35(28-43) vs. 36(29-45)) and SAPS II (4(2-6) vs. 4(2-7)) in the OND-medication group.

### CMA revealed that inflammation as a potential mediator

**Figure 3:**
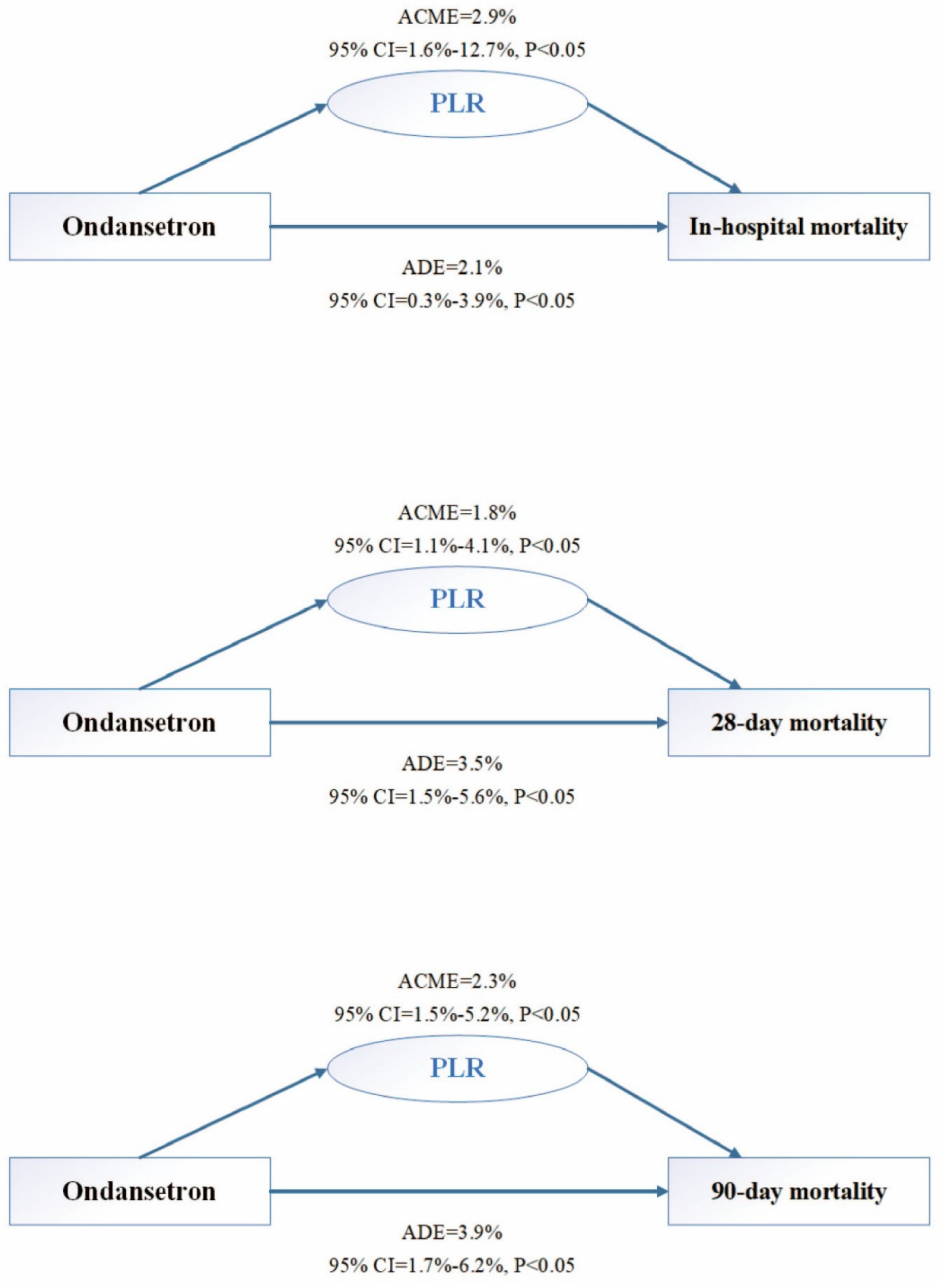
** Causal mediation analysis.** (A) The causal mediation relationship for in-hospital mortality after adjusting by age and gender in study participants. (B) The causal mediation relationship for 28-day hospital mortality after adjusting by age and gender in study participants. (C) The causal mediation relationship for 90-day hospital mortality after adjusting by age and gender in study participants. ADE, average direct effect. ACME, average causal mediation effect. CI, confidence interval.

Previous studies have demonstrated that PLR is a great prognostic predictor of CVD, including MI^19^,^20^. Interestingly, as presented in **Figure 3(A)**, a direct effect was observed between early OND treatment and in-hospital mortality, accounting for 2.1% (95% CI: 0.3-3.9%). As for the mediator, PLR could explain 2.9% (95% CI: 1.6-12.7%) of the impact of OND use on in-hospital mortality after adjusting for age and sex. Similarly, the causal mediation relationship was significant for 28-day mortality and 90-day mortality (**Figure 3(B),(C)**). The ADEs of OND medication on 28-day and 90-day mortality of patients with MI were 3.5% (95% CI: 1.5-5.6%) and 3.9% (95% CI: 1.7-6.2%), and PLR could explain 1.8% (95% CI: 1.1-4.1%) and 2.3% (95% CI: 1.5-5.2%) for the total effect, respectively.

### Sensitive analysis

A moderate confounder (ρ = 0.1) could turn this indirect effect into zero (**Supplemental Figure 1**). Regarding CMA, sensitivity analysis revealed that the indirect effect between early OND medication and in-hospital mortality was relatively reliable.

## Discussion

In this study, we investigated the previously unrecognized role of OND in reducing mortality risk in critically ill patients with MI, including reducing in-hospital, 28-day, and 90-day mortality. In addition, we used CMA to further investigate the potential mechanism and observed that the protective effect of OND on patients with MI was at least partly dependent on its anti-inflammatory activity. Thus, our data provided a novel therapeutic option for critically ill patients with MI, which required further clinical trials.

OND is widely used in clinical practice to treat nausea and vomiting, especially in patients undergoing chemotherapy. Recently, the anti-inflammatory effect of OND in inflammation-related diseases attracted the attention of researchers. For instance, OND medication improved stool consistency, frequency, urgency, and bloating in patients with irritable bowel syndrome (IBS)^21^,^22^. Interestingly, the positive effect in patients with IBS was even higher in those with baseline C-reactive protein above the median (2.09 mg/L)^23^ suggesting that OND may interact with low-grade inflammation. Similarly, an association was observed between the use of OND and reduced mortality in patients with mechanical ventilation by modulating inflammation^24^. Taken together, these studies revealed that OND has a broad indication and may have a potent anti-inflammatory effect, which may benefit critically ill patients with MI.

Inflammation is a major cause of adverse patient outcomes post-MI^25^. Aberrant activation of neutrophils was proved to be critically involved in several inflammatory diseases, including ischemia-reperfusion injury^26^-^28^. Neutrophils were recruited to cardiac tissue^27^,^28^ and worsened myocardial injury via inducing cardiomyocyte death 29,30. Moreover, suppressing myocardial inflammation was found to be beneficial post-MI^10^. To further reveal the underlying mechanisms and causality of the protective effect of OND in patients with MI, we hypothesize that targeting inflammation plays a mediating role for OND in the treatment of MI. Growing evidence has shown that PLR is reliable as a marker of systemic inflammation in patients^31^,^32^. In this study, we observed that OND was a potential causal factor in the regulation of the PLR affecting survival in patients with MI. These data demonstrated that OND might alleviate systemic inflammation in patients with MI to improve their prognosis.

Serotonin and 5-HT signaling could modulate the immune system^33^, which might explain the anti-inflammatory effect of OND as a 5-HT_3_ receptor antagonist. Increased circulating levels of serotonin were observed in patients with post-MI and those with complex coronary lesions^34^,^35^. Elevated 5-HT levels post-MI may be associated with platelet activation, mood disorders, and thrombosis^36^,^37^. Cycloheximide (5-HT_2_A and 5-HT_2_B antagonists) administered after lower extremity MI reduced 5-HT levels while preventing valvular fibrotic remodeling was associated with increased mitral valve size and reduced mitral regurgitation^38^. Additionally, SOFA score was an independent predictor of mortality in patients with myocardial injury^39^. In this study, lower SOFA and SAPS II scores were observed in the OND-medication group, indicting OND might exert its protective effect through relieving disease severity in patients with MI. Taken together, these studies suggested that targeting 5-HT to alleviate systemic inflammation may be a feasible strategy to improve prognosis in patients with MI and OND could be the potential option.

The limitations of this study are as follows. First, this is a retrospective study, further randomized controlled trials are required to prove the reliability of the results. Additionally, although we used various ways including PSM analysis to eliminate potential confounders, some unmeasured confounders might still affect the robustness of the results, especially the mediation effect of inflammation. Hence, more animal experiments and clinical experiments to verify the anti-inflammatory mechanism of OND in MI disease are required.

## Conclusion

Among critically ill patients with MI, early OND treatment may play a protective role in reducing in-hospital, 28-day, and 90-day mortality. Furthermore, CMA demonstrated that the beneficial effect of OND on patients with MI was exerted through an anti-inflammatory effect, at least in part.

## Supplementary Material

Supplementary figure.

## Figures and Tables

**Table 1:** Baseline characteristics of study participants.

	All patients (n=4486)	Non-ondansetron medication (n=3510)	Ondansetron medication (n=976)	P-value
Age (years)	73(63-82)	73(64-82)	72(63-82)	0.976
Male (n (%))	2864(63.8)	2284(65.1)	580(59.4)	0.001
Weight (Kg)	79.5(67.1-93.2)	79.6(67.2-93.6)	79.0(67.0-91.5)	0.362
**Vital signs**				
RR (/min)	19(17-21)	19(17-21)	18(16-20)	<0.001
SBP (mmHg)	115(106-126)	114(105-126)	116(106-127)	0.200
DBP (mmHg)	60(53-67)	60(53-67)	59(53-66)	0.071
Temperature (℃)	36.7(36.5-37.0)	36.7(36.5-37.0)	36.7(36.5-37.0)	0.267
HR (/min)	77(67-86)	76(66-85)	78(68-87)	0.368
**Comorbidities**				
Cerebral hemorrhage (n (%))	138(3.1)	103(2.9)	35(3.6)	0.295
AF (n (%))	446(9.9)	363(10.3)	83(8.5)	0.091
COPD (n (%))	354(7.9)	294(8.4)	60(6.1)	0.022
CKD (n (%))	1733(38.6)	1402(39.9)	331(33.9)	0.001
AKI (n (%))	2083(46.4)	1703(48.5)	380(38.9)	<0.001
CHF (n (%))	2285(50.9)	1844(52.5)	441(45.2)	<0.001
**Clinical data**				
Lymphocyte (%)	17.5(10.4-25.3)	17.7(10.5-25.5)	16.8(9.9-24.5)	0.762
RBC (m/uL)	3.6(3.2-3.9)	3.58(3.25-3.85)	3.57(3.21-3.93)	0.835
WBC (K/uL)	8.7(6.5-11.7)	8.6(6.5-11.5)	9.0(6.8-12.1)	0.371
Platelet (K/uL)	214(174-246)	215(177-246)	214(165-249)	0.602
Creatinine (mg/dL)	1.3(0.9-1.6)	1.4(0.9-1.6)	1.2(0.8-1.6)	0.085
Glucose (mmol/L)	135(116-169)	135(115-173)	135(119-162)	0.806
PLR	121.6(76.5-213.0)	120.7(77.2-211.2)	125.4(74.4-219.5)	0.025
Vasopressin (n (%))	469(10.5)	393(11.2)	76(7.8)	0.002
Diuretic use (n (%))	3615(80.6)	2836(80.8)	779(79.8)	0.493
**Clinical outcomes**				
In-hospital mortality (n (%))	324(7.2)	269(7.7)	55(5.6)	0.030
28-mortality (n (%))	472(10.5)	396(11.3)	76(7.8)	0.001
90-mortality (n (%))	551(12.3)	461(13.1)	90(9.2)	0.001
SAPS II	36(29-45)	36(29-45)	35(28-43)	<0.001
SOFA	4(2-7)	4(2-7)	4(2-6)	<0.001

RR, respiratory rate; HR, heart rate; SBP, systolic blood pressure; DBP, diastolic blood pressure; AF, atrial fibrillation; COPD, chronic obstructive pulmonary disease; CKD, chronic kidney disease; AKI, acute kidney injury; CHF, chronic heart failure; RBC, red blood cell; WBC, white blood cell; SOFA, Sequential Organ Failure Assessment; SAPS II, Simplified Acute Physiology Score II.

**Table 2:** Association between OND treatment and clinical outcomes using multivariate regression analysis.

	HR for 28-day mortality	HR for 90-day mortality	OR for in-hospital mortality
Model 1	0.71(0.56-0.92)	0.72(0.57-0.91)	0.72(0.53-0.97)
Model 2	0.72(0.56-0.92)	0.72(0.57-0.91)	0.72(0.53-0.97)
Model 3	0.71(0.55-0.92)	0.73(0.58-0.92)	0.67(0.49-0.91)

**Model 1** was unadjusted. **Model 2** was adjusted by age and gender. **Model 3** was adjusted by age, gender, congestive heart failure, atrial fibrillation, COPD, CKD, cerebral hemorrhage, white blood cell, red blood cell, glucose, creatinine, platelet. OR, odds ratio; HR, hazard ratio.

**Table 3:** Baseline characteristics of study participants after propensity score matching.

	All patients (n=1948)	Non-ondansetron medication (n=974)	Ondansetron medication (n=974)	P-value
**Matched items**				
Age (years)	73(64-82)	74(64-82)	72(63-82)	0.606
Male (n (%))	1147(58.9)	567(58.3)	580(59.6)	0.580
Weight (Kg)	78.6(66.6-91.6)	77.7(66.4-91.9)	79.1(67.0-91.6)	0.467
SBP (mmHg)	115(106-126)	115(106-126)	116(106-127)	0.985
DBP (mmHg)	59(53-67)	60(53-67)	59(53-66)	0.291
RR (/min)	18(16-21)	18(16-21)	18(16-20)	0.461
Temperature	36.7(36.5-36.9)	36.7(36.5-36.9)	36.7(36.5-36.9)	0.075
CHF (n (%))	859(44.1)	418(43.0)	441(45.3)	0.315
CKD (n (%))	655(33.7)	324(33.3)	331(34.0)	0.773
AKI (n (%))	746(38.3)	366(37.6)	380(39.1)	0.544
AF (n (%))	178(9.1)	95(9.8)	83(8.5)	0.387
Cerebral hemorrhage(n(%))	67(3.4)	33(3.4)	34(3.5)	1.000
Lymphocyte (%)	17.1(10.2-24.9)	17.6(10.5-25.1)	16.8(10.0-24.6)	0.535
RBC(m/uL)	3.6(3.2-3.9)	3.6(3.2-3.8)	3.6(3.2-3.9)	0.929
WBC(K/uL)	8.7(6.6-11.7)	8.5(6.4-11.3)	9.0(6.8-12.2)	0.589
platelet (K/uL)	213.3(170.0-247.0)	212.8(173.4-246.0)	213.9(165.7-249.2)	0.647
creatinine(mg/dL)	1.2(0.8-1.6)	1.3(0.9-1.6)	1.2(0.8-1.6)	0.599
glucose(mmol/L)	136(118-167)	136(116-171)	136(119-163)	0.594
Vasopressin (n (%))	166(8.5)	90(9.2)	76(7.8)	0.291
Diuretic use (n (%))	1536(78.9)	760(78.1)	776(79.8)	0.404
**Clinical outcomes**				
In-hospital mortality (n (%))	133(6,8)	78(8.0)	55(5.7)	0.048
28-day mortality (n (%))	181(9.3)	105(10.8)	76(7.8)	0.024
90-day mortality (n (%))	212(10.9)	122(12.5)	90(9.2)	0.024
SAPS II	35(28-44)	36(29-45)	35(28-43)	0.003
SOFA	4(2-7)	4(2-7)	4(2-6)	0.016

RR, respiratory rate; SBP, systolic blood pressure; DBP, diastolic blood pressure; AF, atrial fibrillation; CKD, chronic kidney disease; AKI, acute kidney injury; CHF, chronic heart failure; RBC, red blood cell; WBC, white blood cell; SOFA, Sequential Organ Failure Assessment; SAPS II, Simplified Acute Physiology Score II.
