# Supplementary material for: Effects of ondansetron treatment on outcomes of critically ill patients with myocardial infarction partly through its anti-inflammatory activity
Source: Int J Med Sci. 2023 Apr 17;20(6):709–16. doi: 10.7150/ijms.81797 (PMC10198140; doi:10.7150/ijms.81797)
Supplement: Supplementary file 1 — Supplementary figure. [file ijmsv20p0709s1.pdf]

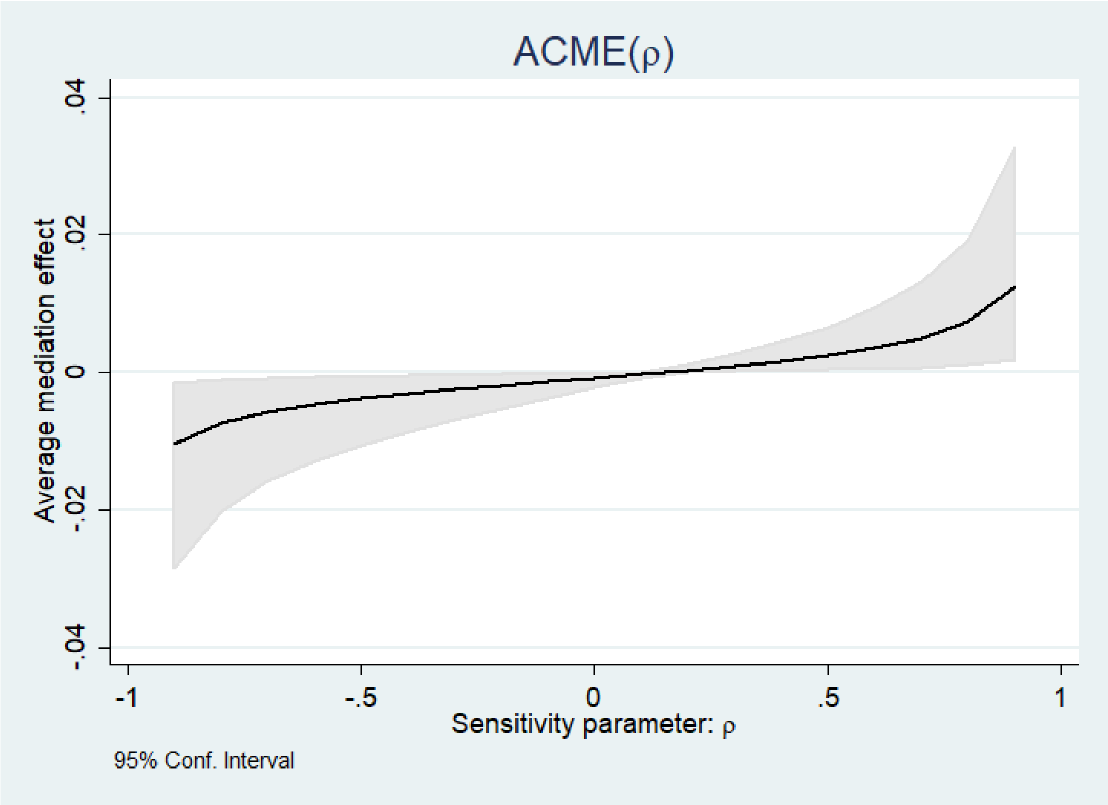

Supplemental Figure 1 Sensitivity analysis of causal mediation analysis for in-hospital mortality
